# Hepatocellular adenomas: is there additional value in using Gd-EOB-enhanced MRI for subtype differentiation?

**DOI:** 10.1007/s00330-020-06726-8

**Published:** 2020-02-21

**Authors:** Timo Alexander Auer, Uli Fehrenbach, Christian Grieser, Tobias Penzkofer, Dominik Geisel, Moritz Schmelzle, Tobias Müller, Hendrik Bläker, Daniel Seehofer, Timm Denecke

**Affiliations:** 1 Klinik für Radiologie, Campus Virchow-Klinikum, Charité – Universitätsmedizin Berlin, 13353 Berlin, Germany; 2 Klinik für Allgemein, Viszeral und Transplantationschirurgie, Campus Virchow-Klinikum, Charité – Universitätsmedizin Berlin, Berlin, Germany; 3 Medizinische Klinik m.S. Gastroenterologie und Hepatologie, Campus Virchow-Klinikum, Charité – Universitätsmedizin Berlin, Berlin, Germany; 4 Institut für Pathologie, Charité – Universitätsmedizin Berlin, Berlin, Germany; 5 Hepatobiliäre Chirurgie & Viszerale Transplantation, Universitätsklinikum Leipzig, Leipzig, Germany; 6 Klinik für Diagnostische und Interventionelle Radiologie, Universitätsklinikum Leipzig, Leipzig, Germany

**Keywords:** Liver, Magnetic resonance imaging, Hepatic neoplasms, Hepatocellular adenoma, Gd-DTPA

## Abstract

**Purpose:**

To differentiate subtypes of hepatocellular adenoma (HCA) based on enhancement characteristics in gadoxetic acid (Gd-EOB) magnetic resonance imaging (MRI).

**Materials and methods:**

Forty-eight patients with 79 histopathologically proven HCAs who underwent Gd-EOB-enhanced MRI were enrolled (standard of reference: surgical resection). Two blinded radiologists performed quantitative measurements (lesion-to-liver enhancement) and evaluated qualitative imaging features. Inter-reader variability was tested. Advanced texture analysis was used to evaluate lesion heterogeneity three-dimensionally*.*

**Results:**

Overall, there were 19 (24%) hepatocyte nuclear factor (HNF)-1a-mutated (HHCAs), 37 (47%) inflammatory (IHCAs), 5 (6.5%) b-catenin-activated (bHCA), and 18 (22.5%) unclassified (UHCAs) adenomas. In the hepatobiliary phase (HBP), 49.5% (39/79) of all adenomas were rated as hypointense and 50.5% (40/79) as significantly enhancing (defined as > 25% intralesional GD-EOB uptake). 82.5% (33/40) of significantly enhancing adenomas were IHCAs, while only 4% (1/40) were in the HHCA subgroup (*p* < 0.001). When Gd-EOB uptake behavior was considered in conjunction with established MRI features (binary regression model), the area under the curve (AUC) increased from 0.785 to 0.953 for differentiation of IHCA (atoll sign + hyperintensity), from 0.859 to 0.903 for bHCA (scar + hyperintensity), and from 0.899 to 0.957 for HHCA (steatosis + hypointensity). Three-dimensional region of interest (3D ROI) analysis showed significantly increased voxel heterogeneity for IHCAs (*p* = 0.038).

**Conclusion:**

Gd-EOB MRI is of added value for subtype differentiation of HCAs and reliably identifies the typical heterogeneous HBP uptake of IHCAs. Diagnostic accuracy can be improved significantly by the combined analysis of established morphologic MR appearances and intralesional Gd-EOB uptake.

**Key Points:**

***•*** *Gd-EOB-enhanced MRI is of added value for subtype differentiation of HCA.*

***•*** *IHCA and HHCA can be identified reliably based on their typical Gd-EOB uptake patterns, and accuracy increases significantly when additionally taking established MR appearances into account.*

***•*** *The small numbers of bHCAs and UHCAs remain the source of diagnostic uncertainty.*

## Introduction

Hepatocellular adenomas (HCAs) are rare benign neoplasms of the liver. The highest incidence is found in young women with a history of oral contraceptive (OCP) use [1–3]. For decades, no subgroup classification of HCAs existed. Since the introduction of the Bordeaux classification in 2006, HCAs have been subdivided into hepatocyte nuclear factor (HNF)-1a-mutated (HHCA), inflammatory (IHCA; formerly known as telangiectatic focal nodular hyperplasia), b-catenin-activated (bHCA), and unclassified (UHCA) HCA [4–12]. This new classification has led to a change in treatment algorithms [4, 12, 13].

In the updated molecular classification of 2017, b-catenin-activated adenomas are furthermore subdivided into an exon 3–mutated and an exon 7–8–mutated subtype. Another subtype has been identified recently and has been termed sonic hedgehog HCA (shHCA) based on its molecular pathway. Previously counted among unclassified adenomas, shHCAs are assumed to account for about 5% of all adenomas [14, 15]. Generally, lesions > 5 cm should be resected because of their increased risk of rupture, bleeding, and malignant transformation. For lesions < 5 cm, treatment is more individually based on the histological subtype. IHCAs have been associated with the presence of hepatic steatosis [16].

Following precise diagnostic characterization, an individual estimate of a patient’s risks and possible complications has to be performed. As HHCAs and small IHCAs are less likely to transform into hepatocellular carcinomas (HCCs), they are typically managed by regular follow-up at intervals of 6–12 months [17]. In general, patients with diagnostically proven HCA should avoid any triggers adenomas are linked with, for instance by intermitting oral contraceptives (OCPs) or steroids and/or by lifestyle modification to lower their body mass index (BMI) [3, 12, 18]. Resection or close follow-up is indicated in patients with bHCA, which is believed to be more prone to malignant transformation and is more often observed in men or patients with, or concomitant glycogen storage disease [15, 16]. Overall, malignant transformation is relatively rare, occurring in 5–10% of all patients, and is most commonly observed in bHCA [15, 16]. Reports in the literature describe bHCA/IHCA mixed types, which tend to be associated with a higher risk of malignancy. In addition, IHCAs in general are associated with a higher risk of bleeding; therefore, patients with these adenomas should also be monitored more closely [8, 15, 16, 19, 20].

Several recent studies have shown the value of magnetic resonance imaging (MRI) with use of a liver-specific contrast agent such as gadobenate dimeglumine (Gd-BOPTA; MultiHance, Bracco Imaging) or gadoxetic acid (Gd-EOB; Primovist or Eovist, Bayer Pharma) in differentiating focal liver lesions (especially FNH and HCA) [19, 21–26]. Gd-EOB seems to be cost-effective for differentiation of malignant and benign liver lesions in routine clinical management [27] and might also have the potential to differentiate HCA subtypes. However, only a few studies have addressed this issue so far. Reliable noninvasive identification of (even small) IHCA or bHCA could change clinical management because of the increased risk of bleeding or malignant transformation of these histological subtypes [8, 16, 20]. Published data suggest that, on hepatobiliary phase (HBP) images, most HCAs are hypointense compared with surrounding liver parenchyma, while a minority of HCAs are iso- or even hyperintense [21, 23, 28]. Conversely, more IHCAs and bHCAs appear to be iso- or hyperintense on HBP images, while HHCAs have been found to have the lowest late-phase intensity [28–30].

The purpose of this study was to investigate the diagnostic value of Gd-EOB-enhanced MRI in differentiating histological subtypes of HCAs.

## Materials and methods

### Patients

Our institutional review board approved this retrospective study (internal registration number EA2/016/14) and waived informed consent due to the retrospective nature. The study protocol conforms to the ethical guidelines of the 2002 Declaration of Helsinki. All patients with histopathologically confirmed HCA who underwent Gd-EOB-enhanced liver MRI between January 2009 and March 2019 were retrospectively identified from the institutional databases.

Only surgically resected lesions were included. A “blinded” pathologist evaluated all macroscopic and microscopic features, which was followed by reprocessing for immunohistochemical analysis. HCAs were classified into the four major molecular subgroups according to their genetic and phenotypic characteristics (HNF-1a-mutated adenoma (HHCA), inflammatory adenoma (IHCA), β-catenin-activated adenoma (bHCA), and unclassified adenoma (UHCA)) [4, 8, 12, 13]. Some of the patients included in the present analysis participated in a previous HCA study that did not include a subgroup analysis [23]. We identified and enrolled a total of 48 patients with 79 HCA lesions. There were 44 female and four male patients. They had a mean age of 38.5 ± 10.5 years (range, 20–67 years).

### Imaging

MRI was performed at 1.5 T or 3.0 T using phased-array body coils. The standard imaging protocols included precontrast T2-weighted (T2w) sequences with and without fat saturation (FS) and T1-weighted (T1w) sequences with and without FS (including in-/opposed-phase technique). After intravenous administration of Gd-EOB (0.025 mmol/kg body weight; manual or automatic injection at a flow rate of approximately 1–2 mL/s, followed by a 40-mL saline flush), multiphase T1w 3D sequences with FS were acquired during breath-hold (arterial phase with a fixed delay of 15 s, portal venous phase with 50-s delay, and transitional phase with 90-s delay). 3D T1w FS imaging was repeated in the hepatobiliary phase 20 min after contrast administration.

### Qualitative analysis

All images were read by two radiologists blinded to the clinical data. The following qualitative parameters were recorded:

Established MRI features:

- number of lesions
- largest axial diameter
- hyperintense rim of the lesion on T2w sequences, the so-called atoll sign
- intralesional fat deposition (i.e., signal drop on opposed-phase images compared with in-phase images)
- presence of a T2w-hyperintense central scar
- presence of hemorrhagic components (i.e., hyperintensities on unenhanced T1w images with and without FS)

**Fig. 1:**
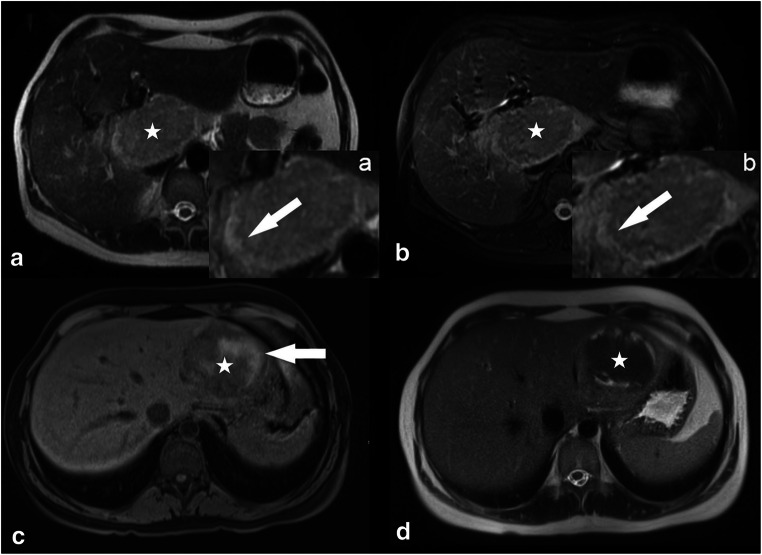
IHCA (**a**, **b**—white stars) showing a classical atoll sign with a typical hyperintense rim in the T2w HASTE and T2 FS sequences (**a**, **b** white arrows). (**c**, **d**) Another patient with IHCA (**c**, **d**—white stars) showing the typical appearance of intralesional bleeding (**c**—white arrow; **c**, non-CE T1w GRE sequence; **d**, T2w HASTE sequence)

Gd-EOB-specific characteristics:

- Readers subjectively rated intralesional Gd-EOB uptake in the HBP as percentage of intralesional iso- to hyperintensity on a 5-point scale (0, 0%; 1, 10–25%; 2, 25–50%; 3, 50–75%; and 4, > 75%) (Fig. 1). Lesions with Gd-EOB uptake scores of 0–1 were classified as “hypointense” and lesions with scores of 2–4 as “significantly enhancing.”

### Quantitative analysis

#### Intralesional Gd-EOB heterogeneity

All lesions were segmented with 3D regions of interest (ROIs) to encompass the whole tumor volume in the HBP using dedicated segmentation software (Medical Imaging Toolkit, MITK). After segmentation, voxel-based texture analysis was performed regarding lesion heterogeneity using the pyradiomics algorithm.

#### Dynamic CE behavior

Polygonal 2D ROIs including the entire tumor at its maximum cross-sectional diameter were placed manually. Cystic and/or hemorrhagic components were spared if reasonably possible. All ROIs were placed in the arterial phase sequence and cloned to the subsequent contrast phase sequences (portal venous and transitional phases). An additional circular ROI with a fixed diameter of 10 mm was placed in healthy liver parenchyma not including vessels and bile ducts. Furthermore, lesion-to-liver enhancement was calculated for the different contrast phases as follows:

$$ \left(100\times \mathrm{lesion}\ \mathrm{enhancement}\right)/\mathrm{liver}\ \mathrm{enhancement}\Big) $$

### Statistics

Statistical analysis was performed with XLSTAT (version 2011.0.01; Addinsoft SARL) and SPSS software (IBM). For the statistical results of the proportional distributions, contingency tables were used. Descriptive parameters are given as mean and standard deviation. Based on histograms and quantile plots, normal distribution was not assumed for metric parameters, and therefore nonparametric tests were performed. Differences in contrast enhancement between HCAs and subtypes were analyzed with a post hoc ANOVA test and Kruskal-Wallis test for paired samples. When possible, Bonferroni-Holm corrections were performed. Cross-tables and the Pearson chi-square test were used to investigate the association of categorical variables. Fisher’s exact test was applied if the cell frequency in a cross-table was less than 5. To test inter-reader correlation, a pairwise two-sided Spearman rank correlation test was performed, and Cohen’s *k* was calculated for inter-reader variability. For calculating the influence of different covariables, a binary logistic regression model was chosen. Diagnostic accuracy in terms of sensitivity and specificity was determined by receiver operating characteristic (ROC) curve analysis. All tests were performed two-sided with a level of significance of 0.05. Boxplots were created with GraphPad Prism (GraphPad Software, Inc.). Voxel heterogeneity was evaluated using texture analysis (pyradiomics).

## Results

### Patients

In 48 patients, a total of 79 pathologically proven HCAs were subdivided into the four groups according to the Bordeaux classification [5] (24% (19/79) HHCAs, 47% (37) IHCAs, 6.5% (5) bHCAs, and 22.5% (18) UHCAs) [4, 12, 13] (Table 1).

**Table 1:** Patient characteristics and EOB MRI findings in a histopathological subgroup analysis of hepatocellular adenomas (HNF-1a-mutated adenoma (HHCA), inflammatory adenoma (IHCA), β-catenin-activated adenoma (bHCA), and unclassified adenoma (UHCA)

	All HCAs (*n* = 79)	HHCA (*n* = 19)	IHCA (*n* = 37)	bHCA (*n* = 5)	UHCA (*n* = 18)	*p* value
Characteristics						
Age	38.5 ± 10.4	38.4 ± 2.5	38.2 ± 1.7	46.4 ± 5.0	37.0 ± 2.2	> 0.05
Gender (female)	61	13	28	4	16	> 0.05
Qualitative analysis						
Lesion diameter (mm)	60.3 ± 36.2	55.8 ± 7.4	57.7 ± 5.9	65.6 ± 30.0	70.1 ± 8.8	> 0.05
Atoll sign (T2w)	21	-	20	-	1	< 0.001
Central scar/septae T2w	9	-	2	3	4	0.002
Steatosis (in/opp)	28	18	6	2	2	< 0.001
Hemorrhage	11	2	3	1	5	> 0.05
Intralesional Gd-EOB uptake						
0–5%	28	15	0	1	12	
5–25%	11	3	4	2	2	
25–50%	13	0	9	1	3	
50–75%	20	1	18	0	1	
> 75–%	7	0	6	1	0	< 0.001
Gd-EOB HBP intensity						
Hypointense	39	18	4	3	14	
Sign. enhancing	40	1	33	2	4	< 0.001
Lesion-to-liver (%) enhancement						
Arterial	399.0 ± 745.1	717.6 ± 1262	255.3 ± 277.5	414.6 ± 442.8	353.8 ± 701.8	0.024
Portal venous	129.5 ± 234.1	275.2 ± 372.3	73.9 ± 181.7	128.0 ± 59.1	90.4 ± 53.5	0.018
Transitional	81.1 ± 96.3	96.4 ± 129.6	73.8 ± 96.8	113.0 ± 35.7	70.5 ± 61.9	> 0.05

### Qualitative analysis

#### Established MRI features

Intralesional steatosis was detected in 95% (18/19) of all HHCAs while it was present in only 17% of the other three subtypes taken together (10/60) (*p* < 0.001) (Fig. 4). A T2w atoll sign was found in 54.0% (20/37) of all IHCAs and in one case of UHCA (*p* < 0.001) (Fig. 1). Our study population included only five bHCA lesions (Fig. 6); 60% (3/5) of these lesions had a central scar on T2w images. Only 8% of the other three subtypes showed a central scar (6/74) (*p* = 0.002) (Table 1). Lesion diameter and intralesional hemorrhage were not found to be significantly different among HCA subtypes (Table 1 and Fig. 1).

#### Gd-EOB MRI features

**Fig. 2:**
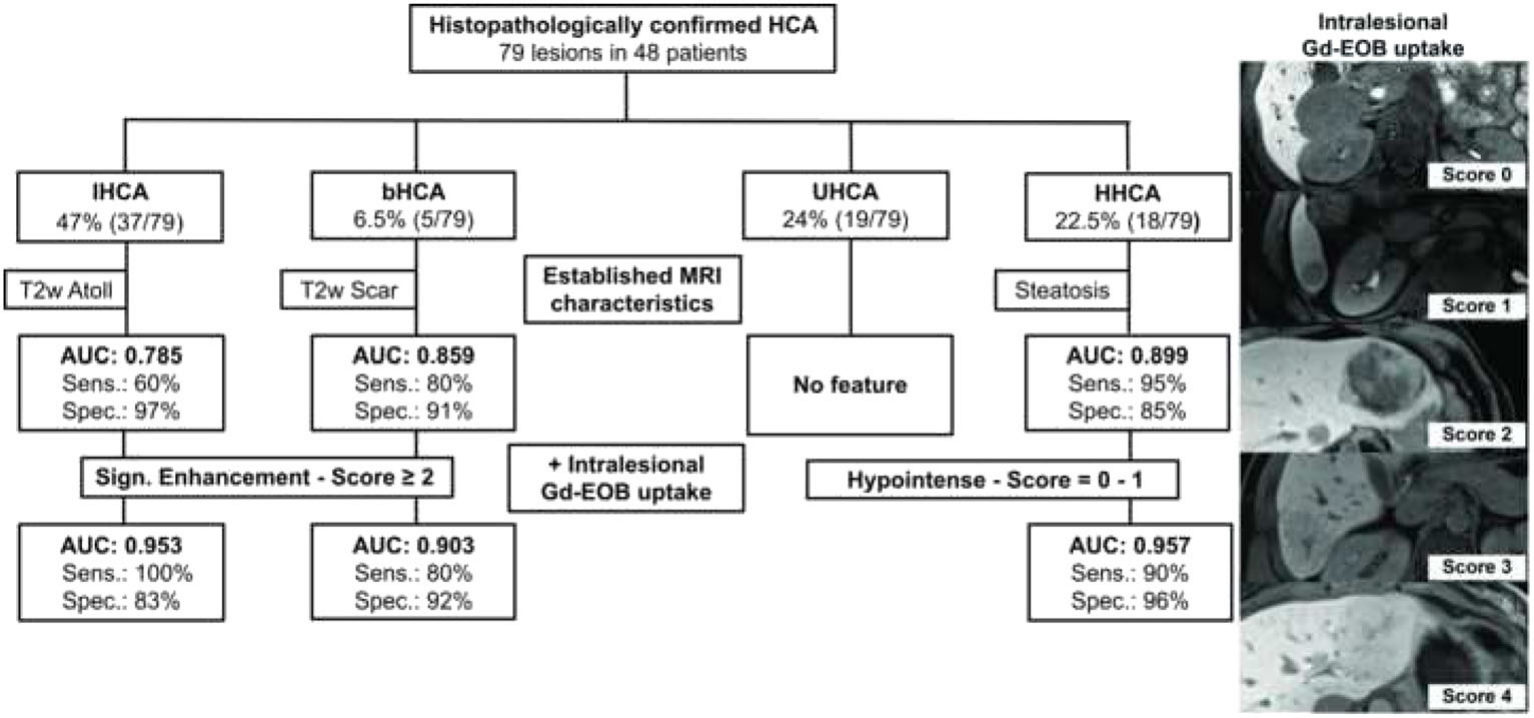
Flowchart of HCA subgroup diagnostic algorithm including combined evaluation of Gd-EOB uptake behavior and established MRI features. Intralesional Gd-EOB uptake in the HBP was rated subjectively as iso- to hyperintensity percentage on a 5-point scale (score 0, 0%; score 1, 10–25%; score 2, 25–50%; score 3, 50–75%; score 4, > 75%)

Intralesional Gd-EOB uptake was rated as intralesional iso- to hyperintensity percentage using a five-step rating system (0, 0–5%; 1, 5–25%; 2, 25–50%; 3, 50–75%; and 4, > 75%) (Fig. 2). In the total study population, 49.5% (39/79) of all adenomas were rated as hypointense (scores of 0–1) and 50.5% as significantly enhancing (40/79) (scores of 2–4) (*p* < 0.001). In the IHCA subgroup, each lesion showed at least an uptake of 5–25%. When a cutoff of at least 25% uptake (scores of 2–4) was applied, 82.5% (33/40) of all IHCAs showed the defined heterogeneous Gd-EOB uptake behavior as opposed to only seven other adenomas (Fig. 6). Accordingly, the IHCA subgroup accounted for 89.0% (24/27) of all adenomas with more than 50% intralesional Gd-EOB uptake (scores of 3–4) in the HBP (*p* < 0.001) (Table 1). In the HHCA subgroup, 95% (18/19) were rated as hypointense (scores of 0–1), while 79% of all HHCAs (15/19) were even rated as homogeneously hypointense (score of 0) without any signs of late phase uptake (*p* < 0.001) (Fig. 5).

#### Combining Gd-EOB-specific and established MRI qualitative characteristics

**Fig. 3:**
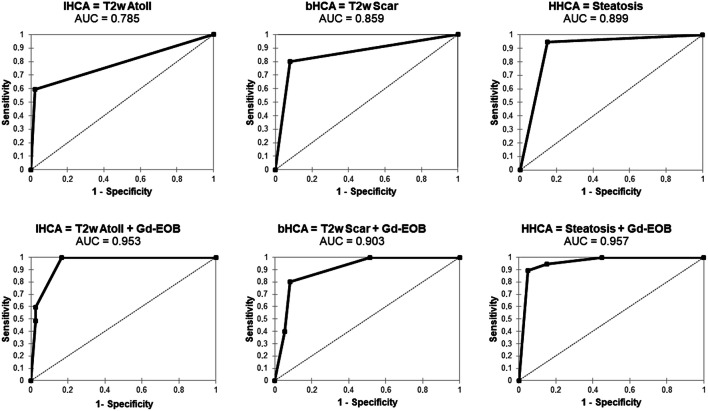
ROC curve analyses. (**a**–**c**) Calculated for IHCA, bHCA, and HHCA and their known established MRI feature. (**d**–**f**) ROC analyses of a binary logistic regression model containing the established MRI feature + the Gd-EOB uptake behavior

When the intralesional Gd-EOB uptake behavior and the presence of a T2w atoll sign were combined, sensitivity increased to 100% while specificity was 83%. Bivariate ROC analysis showed an area under the curve (AUC) of 0.953. The combination of significant uptake and a central scar resulted in 80% sensitivity and 92% specificity for the identification of bHCA (AUC, 0.903). Intralesional Gd-EOB hypointensity combined with intralesional steatosis had 90% sensitivity and 96% specificity for HHCA (AUC, 0.957) (Figs. 2 and 3).

### Quantitative analysis

#### Intralesional Gd-EOB heterogeneity

Volumetric segmentation in the HBP with subsequent texture analysis was performed in 34 patients. Fourteen patients had to be excluded. To strengthen our subjective results of intralesional Gd-EOB heterogeneity in IHCA, we divided the population into “IHCA” and “non-IHCA”. Volume-based analysis revealed significantly increased voxel heterogeneity for the IHCA group (variance of mean SI, 6465.48) compared with the non-IHCA group (variance of mean signal intensity (SI), 2861.80) (*p* = 0.038).

#### Dynamic CE behavior

**Fig. 4:**
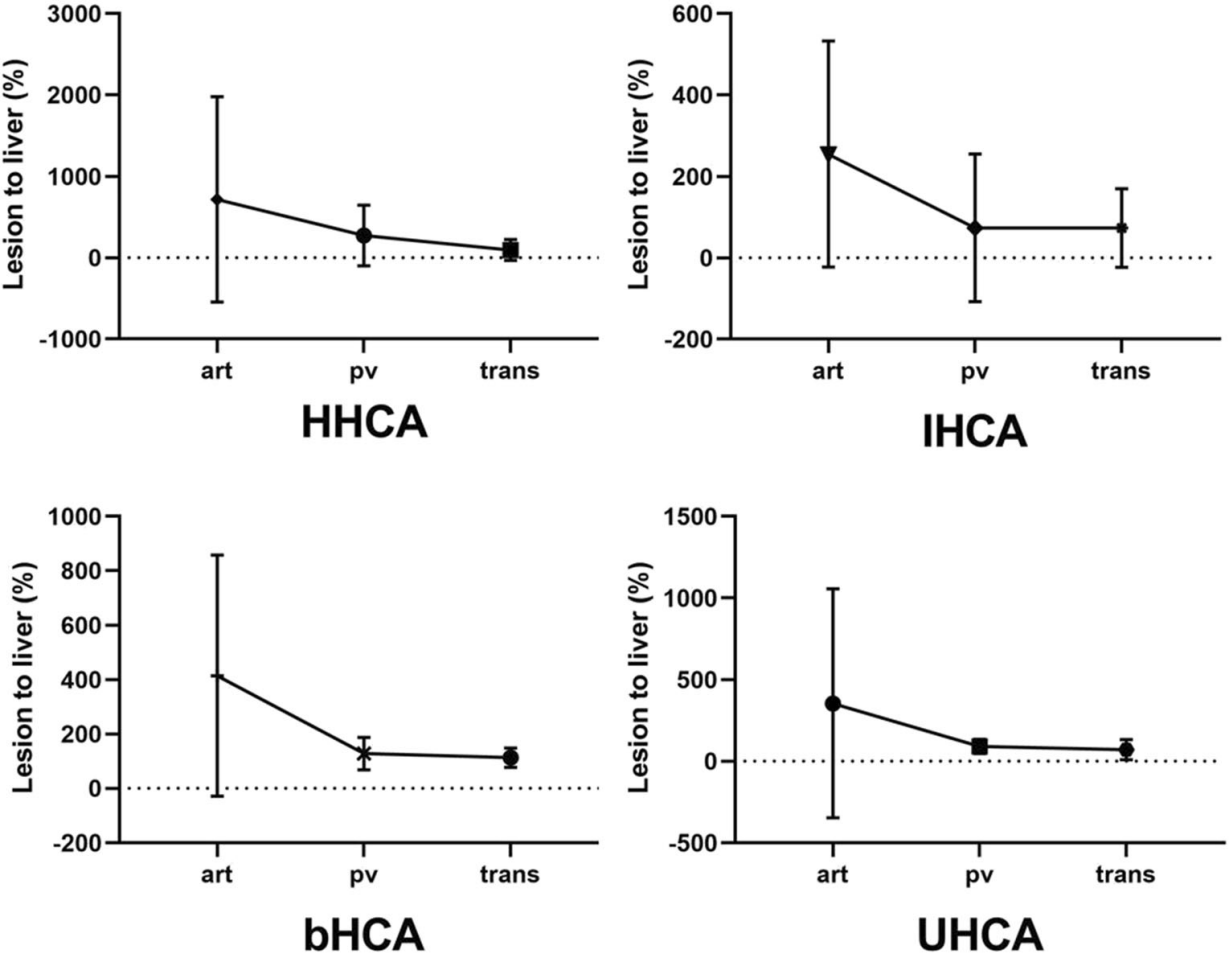
Relative lesion-to-liver (%) enhancement in the arterial (art), portal venous (pv) and transitional (trans) phases for the four subgroups of HHCA, IHCA, bHCA, and UHCA. Kruskal-Wallis test showed significant differences for the arterial (*p* = 0.024) and portal venous (*p* = 0.018) phases and nonsignificant differences for the transitional phase (*p* > 0.05)

ROI-based relative lesion-to-liver enhancement in the late phase was not found to differ significantly between subgroups (*p* = 0.490). For the phases, the Kruskal-Wallis test was significant for the arterial (*p* = 0.024) and portal venous (*p* = 0.018) phase, showing increasingly more marked enhancement for HHCAs compared with the other subtypes (arterial phase, HHCA 717.6 ± 1262 vs. 225 ± 227.5–414.6 ± 442.8 and portal venous phase, HCA 275.2 ± 372.3 vs. 79.9 ± 181.7–275.2 ± 372.3). The Kruskal-Wallis test was negative for the venous phase (*p* = 0.159) (Fig. 4).

## Discussion

Our results show that Gd-EOB MRI provides additional information for differentiating HCA subtypes. IHCAs can be differentiated reliably based on their typical heterogeneous HBP uptake behavior defined in our study as significant enhancement. Furthermore, when Gd-EOB uptake behavior is considered in conjunction with established morphologic MRI features (intralesional steatosis in HHCA, atoll sign in IHCA, and central scar in bHCA), diagnostic accuracy increases significantly. HHCA can be identified with nearly the same reliability as IHCA. However, the small numbers of bHCAs and UHCAs remain a source of diagnostic uncertainty.

In a meta-analysis published in 2017, Guo et al described a low signal intensity (SI) for HCAs in the HBP and concluded that combining SI in the HBP with established MRI features and risk factors of liver disease might improve diagnostic yield in the detection and differentiation of HCAs. Guo et al further considered the benefit of Gd-EOB-enhanced imaging to be overrated [31].

The established MRI characteristics known so far were also present in our study. For instance, HHCAs showed a signal drop in the unenhanced opposed-phase series (95%; *p* < 0.001) consistent with diffuse intralesional steatosis. This is in line with Aalten et al (78%), Laumonier et al (87%), and Tse et al (100%), underlining the accuracy of this simple visual diagnostic sign (28, 32, 33). In 54% of IHCA lesions in our study, the atoll sign was present, yielding nearly 100% specificity for this feature as only one other adenoma also showed the atoll sign (Figs. 2 and 3 and Table 1 [32, 33].

**Fig. 5:**
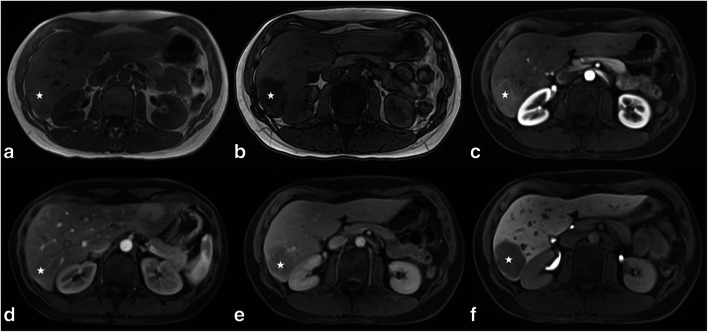
HHCA (white stars) shows isointense signal in the IN-phase image (**a**) with a strong drop in signal in the OPP-phase image indicating presence of lipids (**b**). **c**–**f** Contrast enhancement behavior in the T1w arterial phase with a mild hyperintense signal to the surrounding liver (**c**), an isointense signal in the portal venous phase (**d**) and mildly hypointense in the transitional phase (**e**). In the HBP (**f**), the lesion appears homogeneously hypointense without a significant uptake (0%)

**Fig. 6:**
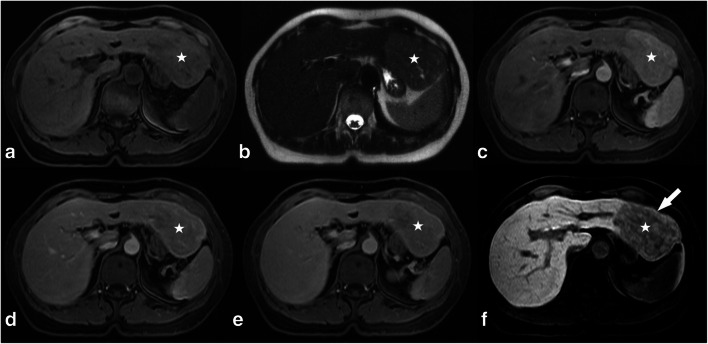
IHCA (white stars) showing isointense signal in the T1w image (**a**) and hyperintensity on standard HASTE image (**b**). **c**–**f** Contrast enhancement behavior in the T1w arterial (hyperintense washin) (**c**), portal venous (heterogeneous washout) (**d**), and transitional (washout) (**e**) phases. In both the portal venous (**d**) and venous phases, the adenoma appears mildly hypointense to the surrounding liver. In the HBP, the lesion shows heterogeneous patchy uptake behavior > 50% and is iso- to hyperintense to surrounding liver (white arrow—(**f**))

In our study population, we noticed a group of adenomas with heterogeneous Gd-EOB uptake in the HBP. Accordingly, we devised a 5-point scale system to subjectively estimate the degree of heterogeneity/level of hyperintensity in the late uptake phase. This was done by two readers blinded to the clinical data, and there was good inter-reader agreement. The enhancement behavior of HHCAs and IHCAs is unequivocal with HHCAs showing essentially no uptake and IHCAs appearing significantly more heterogeneous because of their uptake (*p* < 0.001) (Figs. 5 and 6). These subjective ratings are supported by our volume-based voxel analysis, which showed IHCAs to be significantly more heterogeneous than the other three subtypes.

In its *Clinical practice guidelines on the management of benign liver tumours*, the European Association for the Study of the Liver (EASL) also states that IHCA may present iso- or hyperintense in the HBP, emphasizing the results of a study by Ba-Ssalamah et al [16, 30]. Ba-Ssalamah et al evaluated the degree of OATP1B1/3 and MRP3 expression, which they found to correlate statistically with Gd-EOB retention and washout in the HBP, resulting in 77% specificity for diagnosing IHCA [16, 30]. In our study, we achieved 93% specificity when combining intralesional Gd-EOB heterogeneity with established MRI criteria.

**Fig. 7:**
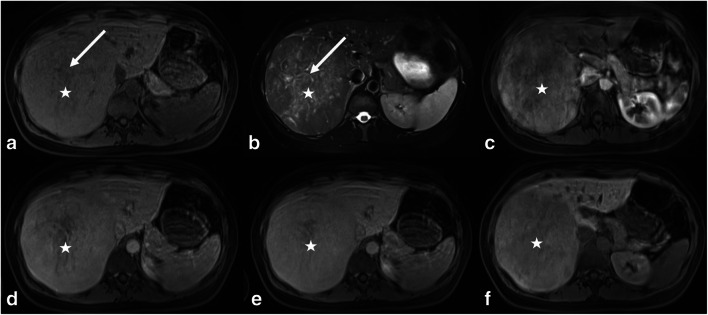
bHCA (white stars) shows isointense signal in the unenhanced T1w VIBE image (**a**) and a central scar/central hypointensity (white arrows) in the T2w FS image (**b**). **c**–**f** Contrast enhancement behavior in the T1w arterial phase with heterogeneous hyperintense washin (**c**), isointensity in the portal venous phase (**d**), and transitional signal intensity to the surrounding liver (**e**). In the HBP, the lesion shows heterogeneous uptake rated as low (0–25%) and is hypointense to the liver

The absence of HBP uptake and subjective impression of hypointensity we found for HHCAs are in line with the results of Tse et al, who found the lowest lesion-to-liver SI ratio for HHCAs and could thus distinguish the latter from other subtypes [28]. In our study cohort, we observed that HHCA and also UHCA can show homogeneous hypointensity in the HBP. Comparison of the two revealed that HHCA appeared more hypointense than UHCA. However, our T1w HBP was acquired with fat saturation so that the even lower signal of HHCAs can be explained by their higher amount of intralesional steatosis. Nevertheless, Tse et al were not able to differentiate more subtypes using Gd-EOB-enhanced imaging [28]. We improved diagnostic performance by categorizing lesions based on intralesional Gd-EOB uptake in the HBP and considering uptake behavior in conjunction with established MRI features. For IHCA (+atoll), bHCA (+scar), and HHCA (+fat), this combined approach resulted in specificities and sensitivities of at least 90%, which was further corroborated by AUC results (Figs. 1 and 2). Even if the results for bHCA lack validity because of the small number of lesions included in the analysis, the diagnostic accuracy reached here is promising (Figs. 1, 2 and 7). Like earlier investigators, we also encountered problems with the unclassified HCA subtype, which does not seem to fit into any pattern.

Besides Gd-EOB-enhanced imaging, a recently published study of Bise et al introduced two new MRI features with high diagnostic accuracy. For IHCAs, Bise et al described the isolated peripheral sinusoidal dilatation “as the crescent sign”, defined as an incomplete hyperintense rim on T2w and/or arterial phase sequences [34]. For HHCAs, Bise et al described a hypovascular pattern; hence, this subtype is hypointense compared with surrounding liver on T1w FS sequences. The lesions with a hypovascular pattern were described as showing mild enhancement in the arterial phase and were found to be characteristically hypointense in the delayed phase [34]. As mentioned in the “Introduction”, the new classification of HCA distinguishes six subtypes. Due to the retrospective character of both ours and Bise et al’s study, MRI features have not yet been tested regarding the new molecular subtypes (bHCA subtypes mutated in exon 3 and 7–8 and shHCA subtype) [34]. Future studies need to evaluate and probably combine Bise et al’s new MRI features and the specific Gd-EOB uptake behavior described by us regarding the new subtypes in order to characterize them noninvasively. Nault et al emphasized the clinical relevance of the new subtypes as bHCAs mutated in exon 3 are more likely to turn into HCC and shHCA are associated with frequent symptomatic bleeding [15]. Nevertheless, given that HCAs are altogether rare, it will take some time to identify enough cases for meaningful analysis.

There are several limitations of the present analysis. First, this is a retrospective study and patients were not enrolled consecutively. Second, as this analysis was restricted to histologically proven HCAs in order to exclude falsely classified lesions from analysis, a selection bias may have occurred. Third, our study is limited by the small number of bHCAs and the small sample size available for voxel-based volumetric analysis. Furthermore, although readers were blinded, they were aware of the study design, which may have introduced detection bias.

In conclusion, combining Gd-EOB uptake behavior in the hepatobiliary phase with established MRI criteria (atoll sign, intralesional fat, and central scar) leads to a high diagnostic accuracy in HCA subtype differentiation. Our approach may improve the noninvasive distinction of IHCA and HHCA and thus help in identifying those patients who might benefit from (early) resection to prevent bleeding or malignant transformation.

## Abbreviations

3D: Three-dimensional
AUC: Area under the curve
bHCA: b-Catenin-activated hepatocellular adenoma
BMI: Body mass index
FNH: Focal nodular hyperplasia
FS: Fat saturation
Gd-BOPTA: Gadolinium benzyloxypropionictetraacetate
Gd-EOB: Gadolinium ethoxybenzyl diethylenetriamine pentaacetic acid
HBP: Hepatobiliary phase
HCA: Hepatocellular adenoma
HHCA: (HNF)-1a-mutated hepatocellular adenoma
IHCA: Inflammatory hepatocellular adenoma
MRP3: Multidrug resistance–associated protein 3
OATP1B1/3: Organic anion–transporting polypeptide 1B3
OCP: Oral contraceptive
ROC: Receiver operating characteristic
ROI: Region of interest
shHCA: Sonic hedgehog hepatocellular adenoma
SI: Signal intensity
T: Tesla
T1w: T1-weighted
T2w: T2-weighted
UHCA: Unclassified hepatocellular adenoma
