# Correction to: Hepatocellular adenomas: is there additional value in using Gd-EOB-enhanced MRI for subtype differentiation?

**DOI:** 10.1007/s00330-021-08386-8

**Published:** 2021-12-05

**Authors:** Timo Alexander Auer, Uli Fehrenbach, Christian Grieser, Tobias Penzkofer, Dominik Geisel, Moritz Schmelzle, Tobias Müller, Hendrik Bläker, Daniel Seehofer, Timm Denecke

**Affiliations:** 1 Klinik für Radiologie, Campus Virchow-Klinikum, Charité – Universitätsmedizin Berlin, 13353 Berlin,, Germany; 2 Klinik für Allgemein, Viszeral und Transplantationschirurgie, Campus Virchow-Klinikum, Charité – Universitätsmedizin Berlin, Berlin, Germany; 3 Medizinische Klinik m.S. Gastroenterologie und Hepatologie, Campus Virchow-Klinikum, Charité – Universitätsmedizin Berlin, Berlin, Germany; 4 Institut für Pathologie, Charité – Universitätsmedizin Berlin, Berlin, Germany; 5 Hepatobiliäre Chirurgie & Viszerale Transplantation, Universitätsklinikum Leipzig, Leipzig, Germany; 6 Klinik für Diagnostische und Interventionelle Radiologie, Universitätsklinikum Leipzig, Leipzig, Germany

**Correction to: European Radiology (2020) 30:3497-3506**

**https://doi.org/10.1007/s00330-020-06726-8**

The article “Hepatocellular adenomas: is there additional value in using Gd-EOB-enhanced MRI for subtype differentiation?”, written by Timo Alexander Auer, Uli Fehrenbach, Christian Grieser, Tobias Penzkofer, Dominik Geisel, Moritz Schmelzle, Tobias Müller, Hendrik Bläker, Daniel Seehofer and Timm Denecke, was originally published Online First without Open Access. After publication in volume 30, issue 6, page 3497–3506 the author decided to opt for Open Choice and to make the article an Open Access publication. Therefore, the copyright of the article has been changed to © The Author(s) 2020 and the article is forthwith distributed under the terms of the Creative Commons Attribution 4.0 International License, which permits use, sharing, adaptation, distribution and reproduction in any medium or format, as long as you give appropriate credit to the original author(s) and the source, provide a link to the Creative Commons licence, and indicate if changes were made.

The images or other third party material in this article are included in the article’s Creative Commons licence, unless indicated otherwise in a credit line to the material. If material is not included in the article’s Creative Commons licence and your intended use is not permitted by statutory regulation or exceeds the permitted use, you will need to obtain permission directly from the copyright holder.

To view a copy of this licence, visit http://creativecommons.org/licenses/by/4.0/.

The original article has been corrected.

